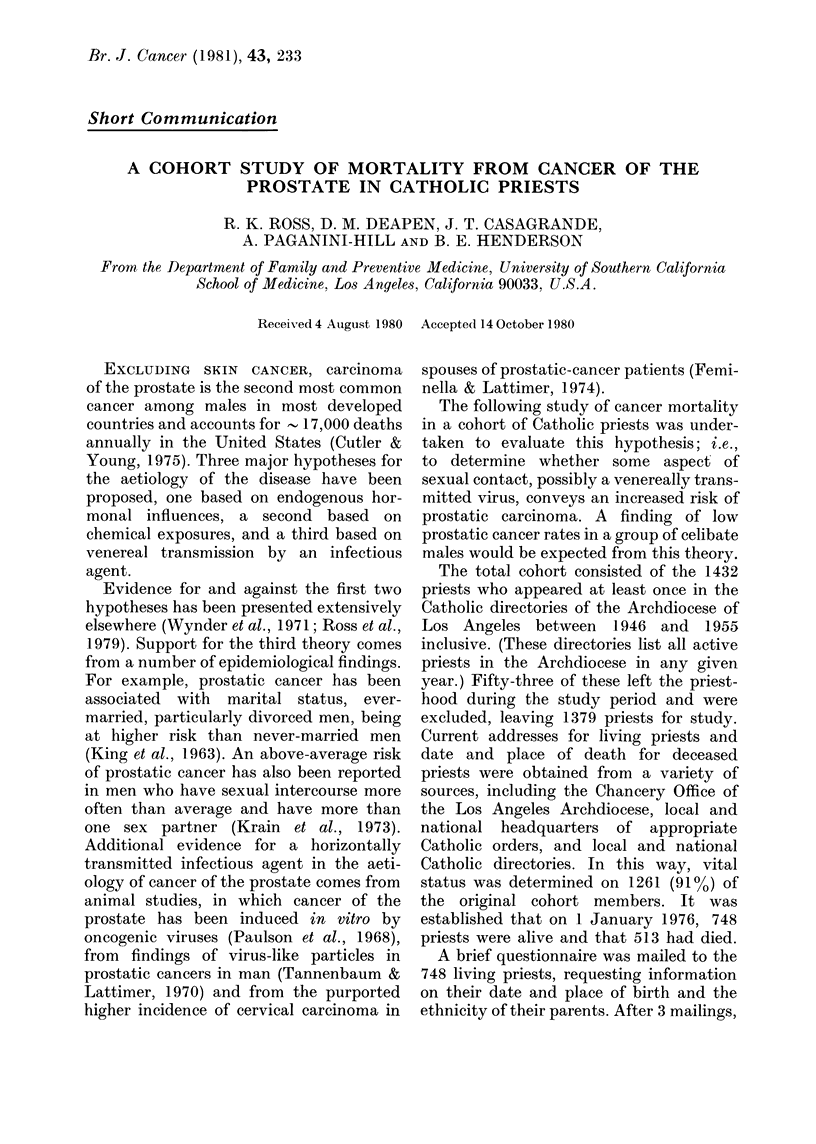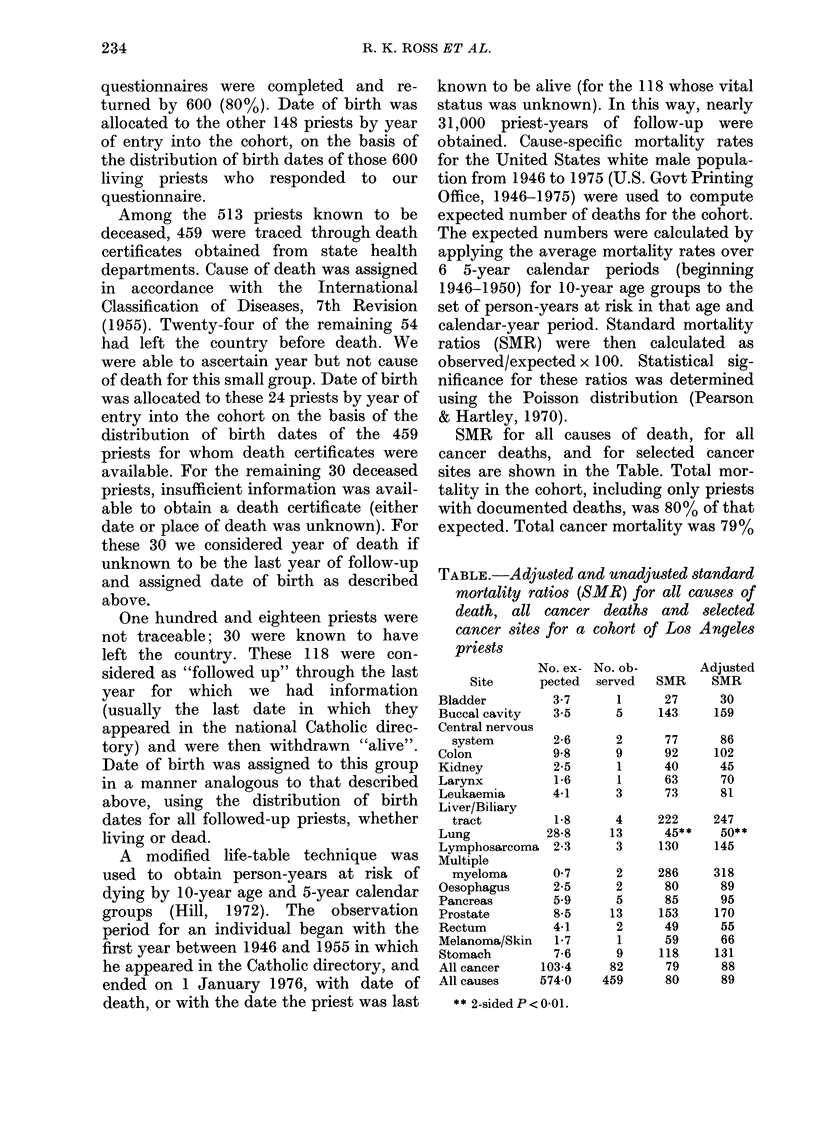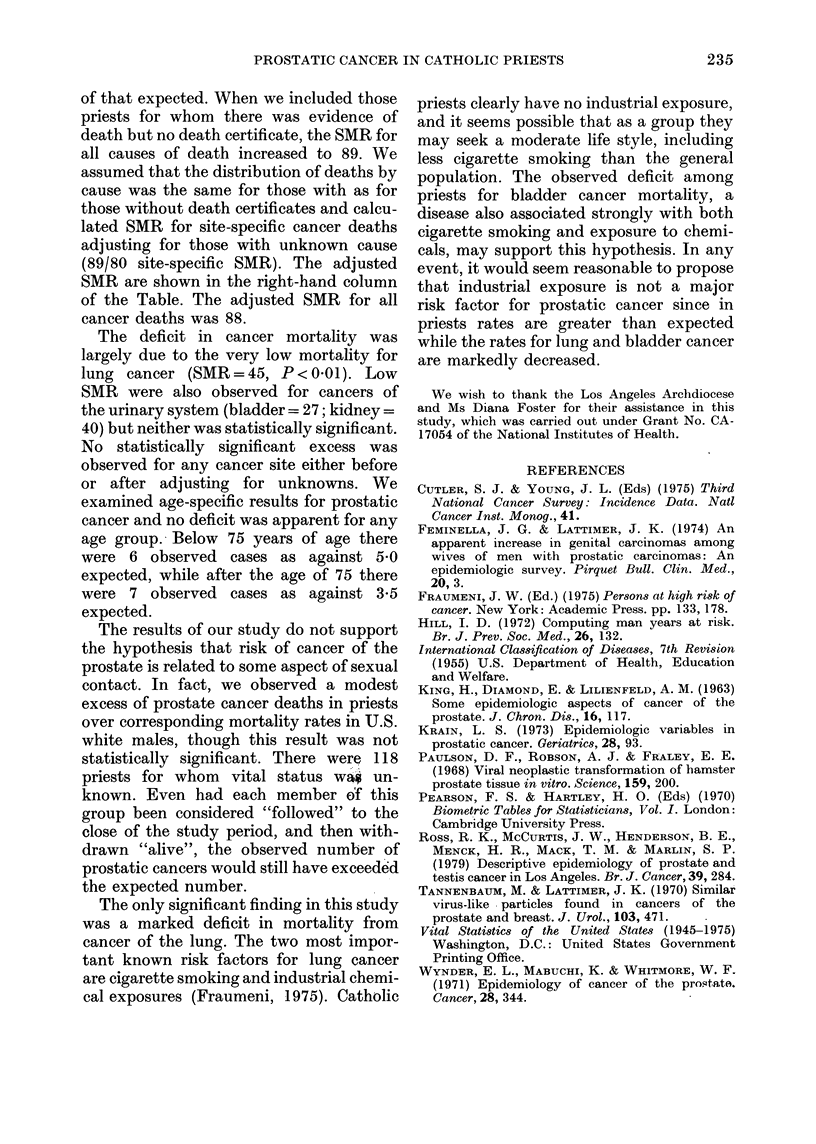# A cohort study of mortality from cancer of the prostate in Catholic priests.

**DOI:** 10.1038/bjc.1981.34

**Published:** 1981-02

**Authors:** R. K. Ross, D. M. Deapen, J. T. Casagrande, A. Paganini-Hill, B. E. Henderson


*Br. J. Cancer* (1981), **43**, 233

*Short Communication*

### A COHORT STUDY OF MORTALITY FROM CANCER OF THE PROSTATE IN CATHOLIC PRIESTS

R. K. ROSS, D. M. DEAPEN, J. T. CASAGRANDE, A. PAGANINI-HILL AND B. E. HENDERSON

*From the Department of Family and Preventive Medicine, University of Southern California School of Medicine, Los Angeles, California* 90033, *U.S.A.*

Received 4 August 1980 Accepted 14 October 1980

EXCLUDING SKIN CANCER, carcinoma of the prostate is the second most common cancer among males in most developed countries and accounts for ~ 17,000 deaths annually in the United States (Cutler & Young, 1975). Three major hypotheses for the aetiology of the disease have been proposed, one based on endogenous hormonal influences, a second based on chemical exposures, and a third based on venereal transmission by an infectious agent.

Evidence for and against the first two hypotheses has been presented extensively elsewhere (Wynder *et al.*, 1971; Ross *et al.*, 1979). Support for the third theory comes from a number of epidemiological findings. For example, prostatic cancer has been associated with marital status, ever-married, particularly divorced men, being at higher risk than never-married men (King *et al.*, 1963). An above-average risk of prostatic cancer has also been reported in men who have sexual intercourse more often than average and have more than one sex partner (Krain *et al.*, 1973). Additional evidence for a horizontally transmitted infectious agent in the aetiology of cancer of the prostate comes from animal studies, in which cancer of the prostate has been induced *in vitro* by oncogenic viruses (Paulson *et al.*, 1968), from findings of virus-like particles in prostatic cancers in man (Tannenbaum & Lattimer, 1970) and from the purported higher incidence of cervical carcinoma in spouses of prostatic-cancer patients (Feminella & Lattimer, 1974).

The following study of cancer mortality in a cohort of Catholic priests was undertaken to evaluate this hypothesis; *i.e.*, to determine whether some aspect of sexual contact, possibly a venereally transmitted virus, conveys an increased risk of prostatic carcinoma. A finding of low prostatic cancer rates in a group of celibate males would be expected from this theory.

The total cohort consisted of the 1432 priests who appeared at least once in the Catholic directories of the Archdiocese of Los Angeles between 1946 and 1955 inclusive. (These directories list all active priests in the Archdiocese in any given year.) Fifty-three of these left the priesthood during the study period and were excluded, leaving 1379 priests for study. Current addresses for living priests and date and place of death for deceased priests were obtained from a variety of sources, including the Chancery Office of the Los Angeles Archdiocese, local and national headquarters of appropriate Catholic orders, and local and national Catholic directories. In this way, vital status was determined on 1261 (91%) of the original cohort members. It was established that on 1 January 1976, 748 priests were alive and that 513 had died.

A brief questionnaire was mailed to the 748 living priests, requesting information on their date and place of birth and the ethnicity of their parents. After 3 mailings, questionnaires were completed and returned by 600 (80%). Date of birth was allocated to the other 148 priests by year of entry into the cohort, on the basis of the distribution of birth dates of those 600 living priests who responded to our questionnaire.

Among the 513 priests known to be deceased, 459 were traced through death certificates obtained from state health departments. Cause of death was assigned in accordance with the International Classification of Diseases, 7th Revision (1955). Twenty-four of the remaining 54 had left the country before death. We were able to ascertain year but not cause of death for this small group. Date of birth was allocated to these 24 priests by year of entry into the cohort on the basis of the distribution of birth dates of the 459 priests for whom death certificates were available. For the remaining 30 deceased priests, insufficient information was available to obtain a death certificate (either date or place of death was unknown). For these 30 we considered year of death if unknown to be the last year of follow-up and assigned date of birth as described above.

One hundred and eighteen priests were not traceable; 30 were known to have left the country. These 118 were considered as "followed up" through the last year for which we had information (usually the last date in which they appeared in the national Catholic directory) and were then withdrawn "alive". Date of birth was assigned to this group in a manner analogous to that described above, using the distribution of birth dates for all followed-up priests, whether living or dead.

A modified life-table technique was used to obtain person-years at risk of dying by 10-year age and 5-year calendar groups (Hill, 1972). The observation period for an individual began with the first year between 1946 and 1955 in which he appeared in the Catholic directory, and ended on 1 January 1976, with date of death, or with the date the priest was last known to be alive (for the 118 whose vital status was unknown). In this way, nearly 31,000 priest-years of follow-up were obtained. Cause-specific mortality rates for the United States white male population from 1946 to 1975 (U.S. Govt Printing Office, 1946–1975) were used to compute expected number of deaths for the cohort. The expected numbers were calculated by applying the average mortality rates over 6 5-year calendar periods (beginning 1946–1950) for 10-year age groups to the set of person-years at risk in that age and calendar-year period. Standard mortality ratios (SMR) were then calculated as observed/expected × 100. Statistical significance for these ratios was determined using the Poisson distribution (Pearson & Hartley, 1970).

SMR for all causes of death, for all cancer deaths, and for selected cancer sites are shown in the Table. Total mortality in the cohort, including only priests with documented deaths, was 80% of that expected. Total cancer mortality was 79%

TABLE.—*Adjusted and unadjusted standard mortality ratios (SMR) for all causes of death, all cancer deaths and selected cancer sites for a cohort of Los Angeles priests*

| Site | No. expected | No. observed | SMR | Adjusted SMR |
|---|---|---|---|---|
| Bladder | 3·7 | 1 | 27 | 30 |
| Buccal cavity | 3·5 | 5 | 143 | 159 |
| Central nervous system | 2·6 | 2 | 77 | 86 |
| Colon | 9·8 | 9 | 92 | 102 |
| Kidney | 2·5 | 1 | 40 | 45 |
| Larynx | 1·6 | 1 | 63 | 70 |
| Leukaemia | 4·1 | 3 | 73 | 81 |
| Liver/Biliary tract | 1·8 | 4 | 222 | 247 |
| Lung | 28·8 | 13 | 45** | 50** |
| Lymphosarcoma | 2·3 | 3 | 130 | 145 |
| Multiple myeloma | 0·7 | 2 | 286 | 318 |
| Oesophagus | 2·5 | 2 | 80 | 89 |
| Pancreas | 5·9 | 5 | 85 | 95 |
| Prostate | 8·5 | 13 | 153 | 170 |
| Rectum | 4·1 | 2 | 49 | 55 |
| Melanoma/Skin | 1·7 | 1 | 59 | 66 |
| Stomach | 7·6 | 9 | 118 | 131 |
| All cancer | 103·4 | 82 | 79 | 88 |
| All causes | 574·0 | 459 | 80 | 89 |

** 2-sided $P < 0·01$.

of that expected. When we included those priests for whom there was evidence of death but no death certificate, the SMR for all causes of death increased to 89. We assumed that the distribution of deaths by cause was the same for those with as for those without death certificates and calculated SMR for site-specific cancer deaths adjusting for those with unknown cause (89/80 site-specific SMR). The adjusted SMR are shown in the right-hand column of the Table. The adjusted SMR for all cancer deaths was 88.

The deficit in cancer mortality was largely due to the very low mortality for lung cancer (SMR = 45, $P < 0{\cdot}01$). Low SMR were also observed for cancers of the urinary system (bladder = 27; kidney = 40) but neither was statistically significant. No statistically significant excess was observed for any cancer site either before or after adjusting for unknowns. We examined age-specific results for prostatic cancer and no deficit was apparent for any age group. Below 75 years of age there were 6 observed cases as against 5·0 expected, while after the age of 75 there were 7 observed cases as against 3·5 expected.

The results of our study do not support the hypothesis that risk of cancer of the prostate is related to some aspect of sexual contact. In fact, we observed a modest excess of prostate cancer deaths in priests over corresponding mortality rates in U.S. white males, though this result was not statistically significant. There were 118 priests for whom vital status was unknown. Even had each member of this group been considered "followed" to the close of the study period, and then withdrawn "alive", the observed number of prostatic cancers would still have exceeded the expected number.

The only significant finding in this study was a marked deficit in mortality from cancer of the lung. The two most important known risk factors for lung cancer are cigarette smoking and industrial chemical exposures (Fraumeni, 1975). Catholic priests clearly have no industrial exposure, and it seems possible that as a group they may seek a moderate life style, including less cigarette smoking than the general population. The observed deficit among priests for bladder cancer mortality, a disease also associated strongly with both cigarette smoking and exposure to chemicals, may support this hypothesis. In any event, it would seem reasonable to propose that industrial exposure is not a major risk factor for prostatic cancer since in priests rates are greater than expected while the rates for lung and bladder cancer are markedly decreased.

We wish to thank the Los Angeles Archdiocese and Ms Diana Foster for their assistance in this study, which was carried out under Grant No. CA-17054 of the National Institutes of Health.

### REFERENCES

CUTLER, S. J. & YOUNG, J. L. (Eds) (1975) *Third National Cancer Survey: Incidence Data. Natl Cancer Inst. Monog.*, **41.**

FEMINELLA, J. G. & LATTIMER, J. K. (1974) An apparent increase in genital carcinomas among wives of men with prostatic carcinomas: An epidemiologic survey. *Pirquet Bull. Clin. Med.*, **20**, 3.

FRAUMENI, J. W. (Ed.) (1975) *Persons at high risk of cancer.* New York: Academic Press. pp. 133, 178.

HILL, I. D. (1972) Computing man years at risk. *Br. J. Prev. Soc. Med.*, **26**, 132.

*International Classification of Diseases, 7th Revision* (1955) U.S. Department of Health, Education and Welfare.

KING, H., DIAMOND, E. & LILIENFELD, A. M. (1963) Some epidemiologic aspects of cancer of the prostate. *J. Chron. Dis.*, **16**, 117.

KRAIN, L. S. (1973) Epidemiologic variables in prostatic cancer. *Geriatrics*, **28**, 93.

PAULSON, D. F., ROBSON, A. J. & FRALEY, E. E. (1968) Viral neoplastic transformation of hamster prostate tissue *in vitro. Science*, **159**, 200.

PEARSON, F. S. & HARTLEY, H. O. (Eds) (1970) *Biometric Tables for Statisticians, Vol. I.* London: Cambridge University Press.

ROSS, R. K., MCCURTIS, J. W., HENDERSON, B. E., MENCK, H. R., MACK, T. M. & MARLIN, S. P. (1979) Descriptive epidemiology of prostate and testis cancer in Los Angeles. *Br. J. Cancer*, **39**, 284.

TANNENBAUM, M. & LATTIMER, J. K. (1970) Similar virus-like particles found in cancers of the prostate and breast. *J. Urol.*, **103**, 471.

*Vital Statistics of the United States* (1945–1975) Washington, D.C.: United States Government Printing Office.

WYNDER, E. L., MABUCHI, K. & WHITMORE, W. F. (1971) Epidemiology of cancer of the prostate. *Cancer*, **28**, 344.